# Neuropathy Dermatitis following Surgical Nerve Injury

**DOI:** 10.1155/2011/234185

**Published:** 2012-01-29

**Authors:** Khalifa E. Sharquie, Adil A. Noaimi, Ali S. Alaboudi

**Affiliations:** ^1^Scientific Council of Dermatology and Venereology, Iraqi Board for Medical Specializations, Medical Collection Office, P.O. Box 61080 Baghdad 12114, Iraq; ^2^Department of Dermatology and Venereology, College of Medicine, University of Baghdad, Baghdad, Iraq; ^3^Department of Dermatology and Venereology, Baghdad Teaching Hospital, Baghdad, Iraq

## Abstract

*Background*. Cutting nerve during operations like saphenous vein grafting and knee joint surgery are common surgical procedures. *Objective*. To report cases of dermatitis at the site of neuropathy following skin incision for saphenous vein grafting and knee joint surgery. *Patients and Methods*. This case report work was done in the Department of Dermatology, Baghdad Teaching Hospital, during 2009-2010, seven cases were recorded, six following saphenous vein grafting and one case after knee surgery. Five males and two females, their ages ranged from 50 to 66 (58 ± 5.033223) years. Detailed history and full clinical examination were done for each patient regarding all points related to their conditions. *Results*. All cases presented around 2-3 months following the operation with dermatitis at the site of operational incision. The dermatitis appeared on one side of the operational scar and at area of neuropathy, and the rash did not cross to contralateral side. The dermatitis was well-defined plaque or patch erythematous slight scaly and nonitchy and subsided within few weeks with or without topical therapy. *Conclusions*. Neuropathy dermatitis is apparently a new variant of dermatitis that follows nerve cut during surgery.

## 1. Introduction

Operative incisions invariably divide numerous cutaneous nerves. In most instances, this is inconsequential, as the terminal branches of these nerves are damaged and there is considerable overlap of the sensory innervations in such areas [1]. Cutting nerve during operations like saphenous vein grafting and knee joint surgery might leave rash few months after the operation; there have been several reported cases of dermatitis at the site of saphenous vein graft scars [2–4], complicating nerve injury after arthroscopic debridement of a medial meniscal cyst [5] and complicating operatively induced anesthetic regions around the knee [1]. Biopsy of this rash showed all features of dermatitis [6].The cause of dermatitis at vein graft sites is unknown. Previous observers have attributed this eruption to localized venous stasis as well as xerosis although objective signs of venous insufficiency were lacking causing neuropathy in the form of saphenous neuralgia which may be associated with vein graft dermatitis. The coexistence of dermatitis and neuropathy and the improvement of both with time suggest there may be an association of the skin changes with loss of sensation in the area [6]. 

So, the aim of the present work was to report cases of dermatitis at the site of neuropathy following skin incision for saphenous vein grafting and knee joint surgery.

## 2. Patients and Methods

In this case report work done in Department of Dermatology and Venereology, Baghdad Teaching Hospital during 2009-2010 seven cases were recorded, six following saphenous vein grafting and one case after knee surgery; five males and two females, their ages ranged from 50 to 66 years with mean ±SD of was  58 ± 5.033223  years.

 A detailed history was taken from each patient regarding age, sex, date of coronary artery bypass grafting (CABG), cutaneous problems (duration, course, site of the lesion, associated symptoms, and treatment), history of drugs taken and other skin or systemic disease. Full dermatological examination was done for each patient.

 Sensory examination was done to asses any sensory loss along the course of the infrapatellar nerve and saphenous nerve using light touch and pin prick test.

 Formal consent was taken from each patient after full explanation about the nature, course, complications, and treatment of his or her problem.

 Ethical approval was obtained from the Scientific Council of Dermatology and Venereology, Iraqi Board for Medical Specializations.

## 3. Results

Seven cases with dermatitis following postsurgical nerve cut were presented. All cases presented around 2-3 months following the operation with dermatitis at the site of operational incision. The dermatitis appeared on one side of the operational scar and at area of neuropathy, and the rash does not cross to contralateral side. The dermatitis was well-defined plaque or patch erythematous slight scaly and nonitchy and subsided within few weeks with or without topical steroid therapy.

**Case 1**

A 58-year-old male developed sudden heart infarction that needed urgent bypass heart surgery. Left saphenous vein was used as graft. The patient was otherwise healthy apart from rash developed three months after coronary artery bypass graft (CABG) on the left leg along the saphenous vein graft incision scar. The rash is nonpruritic and red in color ranging in size from pinpoint to 2 cm in diameter. This gradually enlarged in size and then slowly resolved without therapy leaving residual pigmentation in few weeks. The rash appears only on one side of the incision but never crosses and appeared at the anesthetic area only. The patient's medications included simvastatin and lisinopril therapy.

**Figure 1:**
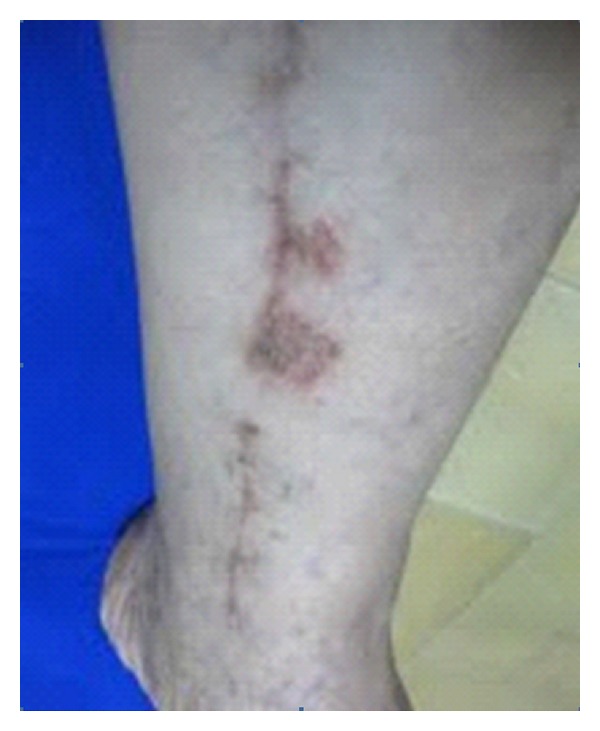
An erythematous discoid scaly patch lateral to the incision scar on the left leg.

On examination, 2 red discoid patches of 15 days duration, scaly, and psoriasiform-like size was 1 cm to 2 cm in diameter (Figure 1). Within few days they started to resolve, losing their scales and becoming dusky red in color. In addition, there are numerous small varicosities along the legs and feet since then there is no recurrence of rash at 1.5-year followup. Biopsy could not be done for fear of developing a leg ulcer. All investigations were normal including blood picture, ESR, and blood biochemistry.

**Case 2**

**Figure 2:**
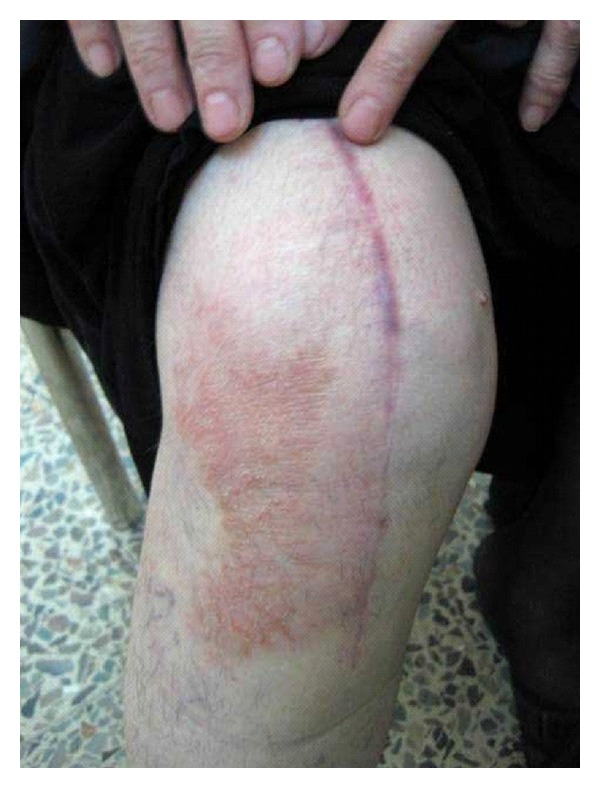
A slightly scaly erythematous plaque on the front of the right knee joint, on the lateral side of the incisional scar only.

A case of 60-year-old women with dermatitis at the lateral side of incision scar following right knee joint replacement about three months postoperatively. She noticed nonitchy rash on the lateral side of the incision scar which had gradually enlarged in size and there was no other important medical history. On examination, slightly scaly erythematous rash formed a plaque on the front of the right knee joint, on the lateral side of the incisional scar only, it did not cross to other side (Figure 2).The rash was completely anesthetic as confirmed by neurological assessment of the infrapatellar nerve (which is branch of saphenous nerve), while there was normal sensation on the medial side of the scar.

**Case 3**

A 66-years-old male developed three months after the saphenous vein grafting in coronary artery bypass graft (CABG) a rash on the left leg along incision scar. Left saphenous vein was used as graft. The rash was nonitchy of four weeks duration; the rash appeared only on one side of the incision but never crossed and appeared at the anesthetic area only. Patient had history of hypertension and diabetes mellitus. The examination revealed erythematous, scaly plaque, with some pigmentation. Patient's medications included aspirin, clopidogrel, and enalapril.

**Case 4**

A 60-years-old female developed three months after CABG a rash on the lateral aspect of right leg anterior to the incision scar of three-week duration. Right saphenous vein was used as graft. The rash was nonitchy of 4-week duration; the rash appeared only on one side of the incision but never crossed and appeared at the anesthetic area only. Patient had history of hypertension and diabetes mellitus. On examination the rash was erythematous slightly crusted and scaly patch. Patient's medications included aspirin, clopidogrel, dilitiazem, insulin, warfarin, and simvastatin.

**Case 5**

A 58-years-old male developed three months after CABG a rash on the right leg of six-week duration. Right saphenous vein was used as graft. The rash was nonpruritic and appeared only on one side of the incision scar but never crossed and appeared at the anesthetic area only. Patient had history of hypertension. On examination, erythematous scaly plaque, leaving pigmentation appeared. Patient's medications included aspirin, clopidogrel, atenolol, warfarin, and atorvastatin.

**Case 6**

A 50-year-old male developed two months after CABG a rash on left leg of four-week duration. Left saphenous vein was used as graft. The rash appears only on one side of the incision scar but never crosses and appeared at the anesthetic area only. Patient had history of hypertension. On examination erythematous scaly plaque was present on left leg lateral to the incisional scar. Patient's medications included aspirin, clopidogrel, atenolol, warfarin, and simvastatin.

**Case 7**

A 54-year-old male developed two months after CABG a rash on right leg of five weeks duration. Right saphenous vein was used as graft. The rash was present only on one side of the incision scar but never crossed to the other side and appeared at the anesthetic area only. Patient had history of hypertension and diabetes mellitus. On examination, red violaceous slightly scaly plaque leaving pigmentation was seen on the lateral aspect of the incisional scar on the anesthetic area. Patient had history of hypertension and diabetes mellitus. Patient's medications included aspirin, clopidogrel, enalapril, and glibenclamide.

## 4. Discussion

This variant of dermatitis appeared at the site of surgical incision that follows saphenous vein harvesting of the leg and the area of knee replacement surgery. The rash appeared around few 2-3 months following the surgery and characterized by well-defined plaque of dermatitis located at the area of sensory loss and at one side of incision and does not cross to other side. 

 The rash is nonitchy and self-limiting and stays for short time usually weeks to a month and then subsideds with or without topical steroid therapy and never recurs again. There are many reports that mentioned dermatitis at the sites of SVH but unfortunately the dermatitis is not well-described and not well-defined as a separate entity as we did in this present report [7–9]. This type of dermatitis has been recently described and the term of neuropathy dermatitis has been suggested [10, 11].

 The mechanism and pathophysiology of this so-called neuropathy dermatitis is difficult to explain but we can speculate that at the time of nerve regeneration there will be release of many neuropeptides from the nerve terminals, and these neuropeptides that are released during nerve regeneration like substance P, calcitonin-gene-related protein (CGRP), vasoactive intestinal peptide (VIP), and neurotensin are involved in the regulation of epidermal antigen presentation [9]. Also these neuropeptides like substance P appear to play a role in both immediate and delayed-type hypersensitivity reactions in the skin. In addition, substance P has been demonstrated to participate in or modulate immediate–type skin hypersensitivity reactions [12].

 In conclusion, neuropathy dermatitis is apparently a new variant of dermatitis that follows nerve cut during surgery. This condition could be attributed to the release of neuropeptides following nerve regeneration.
